# Subtle Gaze and Pupil Dynamics: Detecting Recognition of Familiar Faces with Moving Serial Visual Presentation

**DOI:** 10.5334/joc.492

**Published:** 2026-03-04

**Authors:** Ivory Y. Chen, Sebastiaan Mathôt, Elkan G. Akyürek

**Affiliations:** 1 Department of Experimental Psychology, University of Groningen, Groningen, The Netherlands

**Keywords:** RSVP, moving serial visual presentation (MSVP), familiar face recognition, task-irrelevant attention, eye movement, pupil dilation

## Abstract

A major limitation of the traditional concealed information test (CIT) is its susceptibility to countermeasures. Rapid serial visual presentation (RSVP) paradigms improve resilience against countermeasures, and pupil dilation has been shown to indicate recognition of personally familiar information in the RSVP paradigm. Here, we introduce a novel variation of the RSVP paradigm, Moving Serial Visual Presentation (MSVP), that aims to improve individual detection by incorporating eye gaze and pupil dilation. We combined serial visual presentation with lateral movements to capitalize on pursuit eye movements and pupil size for detecting involuntary recognition and goal-driven suppression of familiar information. Across two experiments, either a target face, a personally familiar face (the participant’s parent), or one of two control faces appeared in a stream. In Experiment 1, participants were required to maintain fixation at a central dot and suppress gaze shifts except toward the task-relevant target face. In Experiment 2, participants were free to move their gaze and responded via keypress. Results indicated that while eye movement measures contributed little to detecting familiar-face processing, both pupil dilation and its rate of change exhibited a noticeable increase in response to familiar faces. At the individual level, classification based on the rate of pupil size change yielded detection rates of 55.2% (Experiment 1) and 33.3% (Experiment 2), exceeding those of previous RSVP-based approaches (22.6%). These findings indicate that MSVP, particularly when eye movements are constrained, enhances the diagnostic value of pupil-based measures for detecting task-irrelevant familiarity, though further work is needed to reach applied standards.

## Introduction

To develop a test of concealed information that a person has in relation to a criminal event has been a long-standing goal in forensic psychology. Achieving this goal requires methods that not only maintain high detection accuracy but also remain robust to countermeasures and are practical enough for real-world use. Because existing approaches vary in their vulnerability to countermeasures, and often rely on equipment or procedures that limit their applicability, there is an ongoing need for simpler, more versatile measures that can reveal meaningful cognitive responses in a reliable way.

At their core, Concealed Information Tests (CITs), originally introduced by Lykken (1959; 1960), attempt to identify concealed knowledge by comparing psychophysiological responses to familiar versus neutral stimuli. In the lab, CITs typically include a mock crime or instructed deception and are designed to capture two key processes that would also apply in the field: the orienting response, which reflects automatic physiological or behavioral reactions triggered by salient or emotionally charged stimuli, and suppression, driven by the motivation to deceive or conceal (Meijer et al., 2016; Verschuere et al., 2011). In typical CIT protocols, a critical comparison is made between probes, which refer to crime-relevant items that only knowledgeable individuals would recognize (e.g., a murder weapon or stolen object), and control items, which are plausible alternatives. When probes elicit more pronounced psychophysiological reactions than control items, it is taken as evidence of recognition of the former (Ben-Shakhar, 2012; Ben-Shakhar & Elaad, 2003; Peth et al., 2016; Bradley & Janisse, 1981; Rosenfeld, 2018).

Beyond mock-crime paradigms, familiar-item CITs extend this framework by using personally meaningful but non-crime-related stimuli, such as one’s own name or a parent’s face, often without requiring deception or concealment (Burton et al., 1999). These paradigms make it possible to study recognition-related orienting and suppression mechanisms under more naturalistic conditions. Importantly, previous research suggests that when the familiar stimuli are highly salient and deeply coded, adding explicit instructions to conceal recognition does not necessarily improve detection accuracy (Ben-Shakhar & Elaad, 2003; Furedy et al., 1994). This indicates that the key cognitive and physiological signatures of familiarity (automatic orienting toward salient stimuli and top-down suppression of attention to them when task-irrelevant) can be examined even in the absence of deliberate concealment.

Here we capitalized on the strengths of the familiar-item paradigm, which we applied in a rapid presentation sequence that should provide resilience against countermeasures, which are deliberate attempts to alter responses to the (control or probe) stimuli to confound the test, and which can drastically reduce the accuracy of the CIT (Ben-Shakhar, 2011, 2012; Peth et al., 2016; Rosenfeld et al., 2004). Our design was inspired by Bowman et al. (2013), who introduced a novel approach that presents stimuli at the edge of awareness, using the Rapid Serial Visual Presentation (RSVP) technique. This method exposes participants to a fast-paced sequence of stimuli, including a familiar item as a probe, as well as control and target items. It is designed to elicit a P3 component from EEG recordings when the meaningful item, the probe, recognized due to its relevance or familiarity, is shown.

An important feature of the CIT-RSVP paradigm is its robustness to countermeasures. Traditional CIT measures are susceptible to deliberate countermeasures that participants can use to confound the results. For example, participants can focus deliberately on control items (Bowman et al., 2014). By presenting items at a rate of 10 per second, the CIT-RSVP paradigms ensure that participants do not have sufficient time to use such countermeasures. This is evidenced by the method’s success in accurately detecting concealed information such as names, even when participants are instructed to use countermeasures such as attempting to elicit strong responses to control items by focusing on them or emotionally engaging with them (Bowman et al., 2014). The CIT-RSVP method has also been proven to be highly effective in detecting famous faces, famous names and online identities with EEG (Alsufyani et al., 2019, 2021; Harris et al., 2021). Although here we do not focus specifically on robustness to countermeasures, it is clearly an important property of any CIT method. For this reason, we take the CIT-RSVP paradigm as a starting point.

The familiar-item RSVP paradigm has also had success by using pupillometry, a relatively easy-to-implement oculomotor measure, as a practical alternative to EEG for detecting responses to personally familiar stimuli. A prior study used pupillometry within the RSVP paradigm to detect recognition of personally familiar faces (probes), while participants were instructed to respond to target faces of the opposite sex. It showed that pupil size can reflect recognition of task-irrelevant familiar faces. However, results have remained modest at the individual level (22.6% detection rate), which severely limits the practical usefulness of the method (Chen, Büchel, et al., 2023). To improve individual detection rates while maintaining the practical advantages of pupillometry over EEG, we considered including additional oculomotor measures. Given that previous investigations into microsaccades and blinks found these measures insufficient for detecting recognition of familiar items in RSVP-based tasks (Chen, Büchel, et al., 2023), the present study investigated whether combining eye-tracking-based indices, especially gaze behaviors and pupil dynamics, can capture the processing of familiar information more sensitively and therefore improve individual detection rates.

To elicit systematic eye movements, we designed a variation of the traditional RSVP paradigm in which stimuli were not presented statically at a central location, but rather moved horizontally. We have dubbed this the Moving Serial Visual Presentation paradigm, which will be introduced in more detail below.

Previous studies have shown that both memory and task demands significantly influence eye movement and gaze behavior. For instance, in sequential face recognition tasks, familiar faces tend to elicit fewer fixations, longer fixation durations, and fewer regions of the face explored compared to unfamiliar faces (Althoff & Cohen, 1999; Hannula et al., 2010; Millen et al., 2017). Similarly, Ryan et al. (2007) demonstrated that when participants were instructed to identify a familiar face, their gaze was preferentially directed toward it within the first second of viewing. In contrast, when participants were instructed to avoid looking at a familiar face, their attention shifted toward unfamiliar faces, but this avoidance behavior emerged more slowly – only after the first second.

Building on such findings, many studies have examined eye movement and gaze patterns as dependent measures in CIT paradigms (Millen & Hancock, 2019; Nahari et al., 2019; Peth et al., 2013). For example, Rosenzweig & Bonneh (2020) showed that involuntary eye movements, particularly microsaccades, can reveal recognition of familiar faces even when presented on the fringe of awareness in a mock terror experiment. Schwedes & Wentura (2012) discovered that when six faces were presented simultaneously, participants fixated longer on concealed familiar faces than on unfamiliar ones, yielding a detection accuracy of 64.9%. Lancry-Dayan et al. (2018) found that participants’ gazes would initially be drawn to a familiar face before shifting to others, enabling reliable detection of concealed knowledge. Similarly, Van Der Cruyssen et al. (2023) applied a mock crime CIT and achieved high classification accuracy by tracking gaze patterns with earlier findings. These results suggest that gaze behavior offers promise as an indicator of concealed recognition, and may be applicable to the CIT-RSVP paradigms as well.

It has been proposed that initial eye movements toward a familiar face – or conversely, the suppression of such movements – may involve both emotional engagement and cognitive control (Rosenfeld et al., 2004; Ryan et al., 2007; Schwetlick et al., 2025). Pupil dilation, in particular, reflects internal states such as emotional arousal (Bradley et al., 2008), processing load (Kahneman & Beatty, 1966), and attentional demands (Gabay et al., 2011). It also indexes the degree of cognitive control required to attend to task-relevant stimuli while inhibiting distractors (Cohen et al., 2015; Querino et al., 2015; Rondeel et al., 2015; van der Wel & van Steenbergen, 2018) and task-irrelevant but salient stimuli (Gilzenrat et al., 2010). These internal changes, whether orienting or suppression, are likely to be captured in pupil dynamics. Therefore, simultaneously monitoring both eye movements and pupil size may be especially powerful: if gaze toward a familiar face is not inhibited, we may detect it through eye movements; if gaze is suppressed, the cognitive control involved may still be observable via increased pupil size.

### The present study

In order to achieve a higher detection rate than the CIT-RSVP paradigms that use only pupil size, here we present an innovative familiar-item paradigm that employs personally familiar faces (specifically, the participant’s parent’s face) as probes within a moving serial visual presentation (MSVP) format. Parental faces were chosen for their high emotional salience and deep familiarity, increasing the likelihood of eliciting strong physiological responses. This choice also reflects the use of salient items in forensic contexts, such as crime-related names or objects, and aligns with our prior RSVP pupillometry study (Chen, Büchel, et al., 2023), allowing direct comparison across different presentation formats.

In this paradigm, three types of stimuli were presented: the probe (the participant’s parent’s face), representing a personally familiar but task-irrelevant stimulus; the control (unfamiliar faces of the same sex), serving as a neutral comparison condition; and the target (faces of the opposite sex), to which participants were instructed to respond, ensuring task engagement. By comparing responses to probes and control faces, we could examine spontaneous orienting and suppression-related physiological signals of familiarity.

Unique to our approach, each face initially appears at the screen center and then moves smoothly to the left or right. Participants were given different instructions across two experiments. In Experiment 1, they were instructed to maintain central fixation and only shift gaze to the target face, requiring accurate eye tracking for a correct response. In Experiment 2, they were free to move their eyes during the trial and responded to the target with a key press afterward. Crucially, Experiment 1 demanded greater control over eye movements, likely engaging more cognitive control than Experiment 2.

Our aim was twofold: first, to assess whether task-irrelevant familiar faces would trigger early gaze attraction and later gaze avoidance – patterns that might reflect orienting responses and task-driven suppression; second, to evaluate whether pupil responses, particularly dilation magnitude and rate of change, reflect suppression demands and enhance individual-level detection, especially under constrained-gaze conditions in Experiment 1. Together, these experiments aim to advance recognition detection by revealing how spontaneous and regulated attention to task-irrelevant salient stimuli can be captured through simple, noninvasive oculomotor measures suitable for practical applications.

## Experiment 1

### Method

#### Participants

Initially, 33 first-year psychology students at the University of Groningen participated in the experiment in exchange for course credits.

Four participants (of 33) were excluded based on low accuracy. Specifically, a response was counted as correct if participants followed the instructed gaze behavior (i.e., gaze shifted > 50 px for targets; remained within 50 px for non-targets) at the moment the critical face disappeared. We used ~70% accuracy as a pragmatic (but not pre-specified) reference point to ensure that participants were able to perform the task at a basic level. The final sample consisted of 29 participants (mean age = 20.3 years, range = 18–24 years, 19 females).

A prior sample size estimation was based on a bootstrap resampling power analysis using data from a previous study that employed the RSVP paradigm to detect participants’ own names (Chen, Karabay, et al., 2023). In that study (N = 31), participants were resampled with replacement, and a linear mixed-effect analysis was performed on each sample using pupil size data within a predefined time window from stimulus onset to 2500 ms (i.e., the period where pupil data were valid and consistently analyzed throughout the study). A ‘hit’ was defined as a significant difference (*p* < .05) between real and control names for more than 5 consecutive samples (200 ms). This process was repeated 1,000 times for each sample size. If more than 900 out of the 1000 iterations produced a hit, the corresponding sample size was considered sufficient (larger than a 90% hit rate across iterations). Based on this criterion, the estimated minimum number of participants required was 25. Given that our current study used the same analysis pipeline and a comparable design, our final sample of 29 participants should be adequately powered. All participants signed up voluntarily and provided written informed consent. In addition, the parents of each participant gave consent and provided a photo of themselves (used as the “familiar face”). All participants reported normal or corrected-to-normal visual acuity and no color blindness. They were instructed to sleep well the night before and refrain from wearing eye makeup. The study was approved by the ethics committee of the Psychology Department of the University of Groningen (approval number: PSY-2023-S-0283) and conducted in accordance with the World Medical Association Declaration of Helsinki (2013).

#### Apparatus and Stimuli

Participants were seated in a dimly lit, sound-attenuated cabin approximately 60 cm from a 27” LCD monitor (Iiyama PL2773H; 1920×1080 pixels; 100 Hz refresh rate). Head position was stabilized using a chin rest and forehead support. Stimuli were presented via OpenSesame 3.3.14 (Mathôt et al., 2012) on the Windows 10 PC. Eye movement and pupil size were recorded monocularly using an EyeLink 1000 system (SR Research) at1000 Hz. A 9-point calibration was conducted prior to the experiment.

During the trials, all face stimuli were displayed against a dark gray background (RGB 40, 40, 40; luminance: 207 cd/m^2^). A white central fixation dot (0.12° × 0.12° visual angle) remained onscreen throughout each trial. Face stimuli, except for the familiar faces, were sourced from two public databases: the 10K US Adult Faces Dataset (Bainbridge et al., 2013) and the Chicago face database (Ma et al., 2015). From both databases, we selected non-celebrity faces with neutral or slight smiles and direct eye contact. No other (demographic) factors were considered for exclusion. Images from the Chicago face database were down-sampled to 200 × 256 pixels to match the resolution of the 10K images. All face images were then standardized using the same pipeline: they were converted to grayscale, adjusted to a mean intensity of 128 (on a 0–255 scale), and cropped with an identical elliptic mask (72 × 100 pixels, or 2.19° × 3.03° visual angle) centered between eyes using the package Imellipse (https://www.mathworks.com/help/images/ref/imellipse.html).

Following standardization, we manually excluded any face images with artifacts (e.g., visible frames around the ellipse, extreme head tilts, closed eyes). The final image pool was divided by age and gender into six subsets: 1. Male Distractors (20–60 years, n = 418), 2. Female Distractors (20–60 years, n = 420), 3. Young Male Targets (≤ 30 years, n = 60), 4. Young Female Targets (≤ 30 years, n = 59), 5. Old Male Targets (≥ 45 years, n = 120), 6. Old Female Targets (≥ 45 years, n = 77).

For the familiar face condition, each participant’s parents submitted an image of themselves (one from the mother and one from the father). The images were required to be high in clarity, with the head occupying approximately 80% of the frame, aligned vertically, facing directly forward with eyes looking straight ahead, and displaying a neutral or mildly smiling facial expression. From these two images, one was randomly selected to serve as the participant’s familiar face stimulus, with the overall set balanced so that the total number of mother and father images was equal across participants. To ensure consistent resolution and appearance across all stimuli, the parental face images were down-sampled to 200 × 256 pixels and processed using the same standardization pipeline as the other face images, including a final manual inspection to verify quality and alignment. The non-familiar stimuli were the same as in our previous RSVP paradigm study, ensuring consistency across paradigms. The collection and standardization of parental faces (used as the familiar stimuli) followed the same procedures as in the earlier work (Chen, Karabay, et al., 2023).

#### Procedure

Each trial presented 13 sequential face images, among which one was a critical face and the remaining 12 were distractor faces. The critical face appeared randomly at the 5^th^, 6^th^, 7^th^, or 8^th^ position, and could be one of four types: a target face, a familiar face, or one of two control faces. These four types occurred equally often (48 trials per condition across 192 total trials), and participants were unaware of which type would appear on any given trial.

The sex of the familiar face determined the sex of all distractor and control faces for a participant (e.g., if the familiar face was the participant’s mother’s face, all distractors and control faces were female; if it was the father, all distractor and control faces were male), while the target face was always of the opposite sex. This design ensured that the familiar face closely resembled distractors and controls in all features except familiarity, while target faces stood out clearly.

The target faces were randomly selected from either the Male Targets or Female Targets, half young and half old, and different in each target trial. The familiar face was the face of the participant’s mother or father, and it remained consistent throughout the experiment (48 trials). To equate presentation frequency, control face 1 and 2 were randomly selected from the Distractors pool (pre-divided into two subsets). These control faces were presented as frequently as the familiar face (48 trials each), allowing for a direct comparison between the two conditions to detect recognition-related effects, while the remaining subset of 400 faces served as a source for sampling 12 unique distractors on each trial. This controlled for low-level visual familiarity and ensured that any observed effects were attributable to personal familiarity.

**Figure 1:**
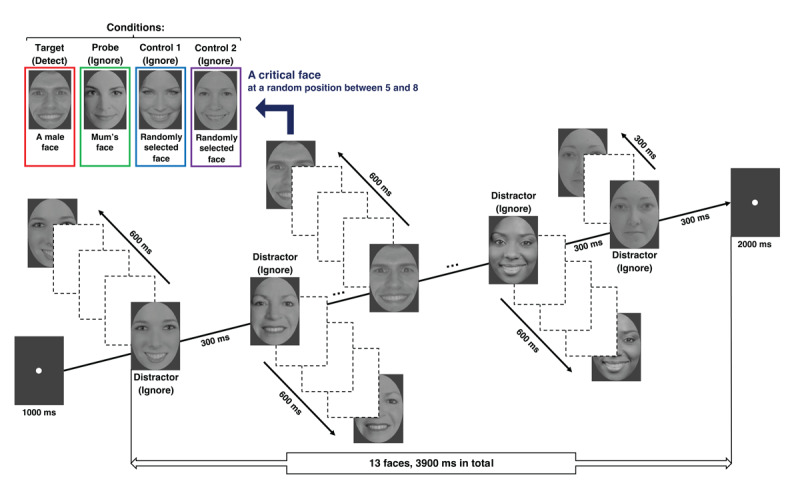
Experimental Trial Sequence Example. *Note*. The short straight arrows indicate the direction of movement for each corresponding image, either horizontally to the left or to the right. The dashed line boxes represent the smooth trajectory along which each image moves. The position of the second identical image along the direction of the short straight arrow marks the final destination and disappearance point of the initial image. This spot is also where participants, upon detecting the target face, were required to focus their gaze to provide their response. Each of the four potential identities of critical faces is represented in distinct colored boxes. For instance, in this example, the target face, highlighted in a red box, depicts a male face next to female distractor faces. The probe face, encased in a green box, is an image of the participant’s mother (actual image not shown for privacy reasons). Meanwhile, the control 1 and control 2 faces, presented in a blue and a purple box separately, are two randomly selected faces. It is important to note that in any given trial, only one critical face was displayed, and its position was pseudo-randomly assigned between the fifth and eighth spot in the stream.

Each trial began with the participants fixating at a central dot. Once stable fixation was detected, the trial began. The fixation dot continued to be displayed until the end of the trial. After 1000 ms, the first face image appeared at the center and started moving smoothly horizontally to the left or right. Every 300 ms, a new face image appeared at the center and began moving horizontally in the opposite direction. Only two face images were visible at a time, and each remained on screen for 600 ms, traveling 60 pixels (approximately 1.83° of visual angle). After all 13 face images had been presented, the fixation dot remained on screen for another 2000 ms to capture any delayed pupil responses. An example of the procedure is illustrated in Figure 1.

Participants were instructed to maintain fixation on the central dot through each trial unless a target face appeared. In that case, they were to move their eyes to follow the target face until it disappeared, then keep their gaze at the last visible location of the target until the end of the trial. For all other faces including familiar and control faces, participants were instructed to ignore them and maintain central fixation. We reasoned that if they would have an involuntary tendency to follow familiar faces, suppressing this tendency would likely require increased cognitive control, potential reflected in greater pupil dilation compared to control faces. Participants completed 24 practice trials (8 each for target and control conditions; familiar faces excluded to avoid habituation). Feedback was provided (green/red dot) after each trial. An accuracy of ≥ 70% was required to proceed the main experiment. If their accuracy was too low, they would be asked to redo the practice until they met the requirement. The main experiment consisted of 192 trials across 12 blocks, with 48 trials for each condition. Summary accuracy feedback was given every 4 blocks, indicating how many of the 64 trials within those blocks participants had correctly performed the target-face detection task. Participants received this numerical feedback as a percentage score, allowing them to monitor their performance and stay motivated. Breaks were provided between blocks.

After the experimental session, participants completed a recognition task. They rated how frequently they had seen 11 face images: their own familiar face, two control faces (each shown 48x), two target faces (once each), two distractor faces (6x), and four faces that were never shown (0x). Responses were on a 5-point scale (0–48 appearances). If a participant assigned a score of 0 to their familiar face, they would be excluded; no such exclusions occurred.

#### Preprocessing of eye-tracking data

The eye-tracking data, encompassing a total of 5568 trials across all participants, was recorded at 1000 Hz and down-sampled to 100 Hz. For each trial, we took the average pupil size of 5 samples prior to the critical face position as a baseline. 211 trials whose baseline pupil size was undefined or where baseline pupil size exceeded 2 standard deviations above or below the mean baseline pupil size were excluded (see Mathôt & Vilotijević, 2023). The remaining trials were simultaneously used for further preprocessing of both eye movements and pupil size. This ensured that subsequent preprocessing and analysis were based on the same dataset and that trials with improper signal recording had already been excluded based on the same criteria.

Our analysis was specifically tailored to assess the horizontal eye movements of participants. To this end, we conducted a preprocessing routine focused exclusively on the horizontal (x-trace) component of the eye movement data, time-locked to the onset of the critical face stimuli. Due to the random positions of critical faces in each trial, samples were distributed unequally at each time point. If the critical faces appeared in the fifth position (the earliest possible position for critical faces, also known as the first critical position), the total time from when the critical faces appeared to when all faces disappeared is 2700 ms. If the critical faces appeared in the eighth position (the last critical position), this duration is 1800 ms. The face presentation was followed by a 2-second fixation period, during which the participants’ eye movements and pupil size continue to be recorded without interruption. Upon examination of the sample distribution over time, we identified a substantial dropout rate in the samples beyond 2500 ms post-stimulus onset. Consequently, we confined our analysis to the initial 2500 ms of data from the x-trace.

To normalize pupil size data, we baselined it by subtracting the average pupil size during the baseline interval from the pupil trace for each trial. We then aligned the pupil trace with the onset of the critical face, ensuring temporal accuracy in our analysis. Consistent with the eye movement data handling, we selected a time frame of 0–2500 ms post-onset for the pupil size data, providing a uniform approach to both sets of measurements.

#### Transparency and Openness

The stimuli (images of familiar faces excluded), data for the experiment and analysis scripts are available on the OSF: https://osf.io/ske56/.

### Results

#### Behavioral data

**Figure 2:**
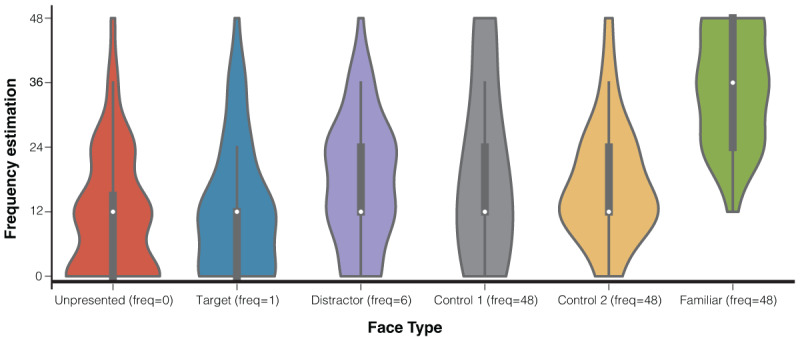
Frequency Estimation in the Memory Task. *Note*. Each color corresponds to a type of face, with the corresponding types and their actual frequencies marked on the X-axis. The vertical black lines in each violin represent the specific estimates given by the participants for the frequency of occurrence of this type of face, while the white dots show the average value.

In Experiment 1, the average accuracy rate (*n* = 29) varied markedly across conditions. For target trials, where participants were instructed to make a gaze shift toward the target face, accuracy was defined as a gaze response in the same direction as the target face and with a horizontal deviation of at least 50 px from central fixation. Under this definition, mean accuracy was 39.1% (range: 4.2–75.0%). This relatively low accuracy may reflect the challenge of detecting the target face while initially fixating at the center, or a failure to execute timely eye movements with sufficiently large deviations of at least 50 px. In contrast, for non-target trials, where participants were instructed to maintain central fixation, accuracy was defined as gaze remaining within 50 px of the fixation dot, regardless of whether the critical face was familiar or one of the control faces. Non-target trials showed high accuracy rates: 95.0% (range: 81.2–100.0%) for familiar-face trials, 95.5% (range: 85.4–100.0%) for Control 1, and 94.6% (range: 81.2–100.0%) for Control 2. To assess whether detection accuracy differed between familiar and control conditions, we compared performance on the familiar condition to the average of the two control conditions using paired t-tests and no significant difference was found (*t*(28) = -0.512, *p* = 0.612), thereby ensuring that subsequent analyses comparing familiar and control conditions were based on trials with statistically equivalent accuracy levels. In the memory task, all participants reported having seen the familiar face as indicated in Figure 2. The estimates for the number of times familiar faces were presented were also higher than the estimates for the number of times other types of faces were presented.

#### Eye movement

Eye movement distance was computed as the horizontal deviation of gaze position from the screen’s midpoint. During each trial, the sign of the distance was determined by the direction of the critical face’s movement: positive values indicate eye movement in the same direction, whereas negative values indicate movements in the opposite direction.

**Group level effect**. To assess group-level effects, we conducted a sample-by-sample linear mixed-effects regression analysis on eye movement distances across conditions (Target, Familiar and the average of Control 1 and Control 2). For every 10 ms within the 2500 ms analysis window, condition was entered as a fixed effect, with the average of the two control conditions serving as the reference. To correct for multiple comparisons, *p*-values across time points were adjusted using the Benjamini-Hochberg (Benjamini & Hochberg, 1995) false discovery rate procedure (*q* = .05), and significant clusters were defined as ≥ 20 consecutive significant samples (≥ 200 ms). This criterion was applied consistently across all sample-by-sample linear mixed effect regression analyses conducted to examine group-level effects in the study.

**Figure 3:**
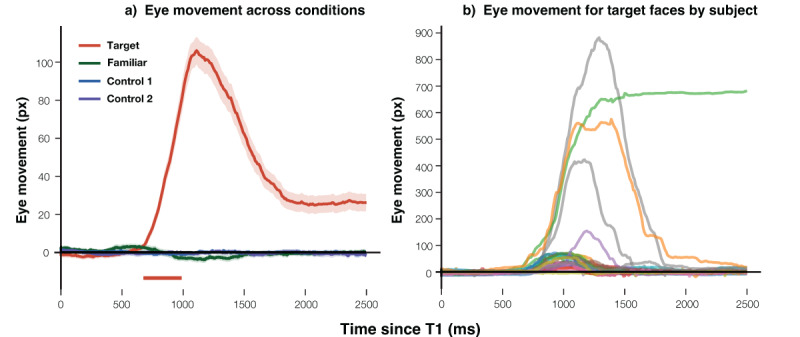
Average Eye Movement Distances. *Note*. **Panel a**) shows the average traces of eye movement distances over time for the four face conditions: the target face (a red line), familiar face (a green line), control 1 face (a blue line), and control 2 face (a purple line). **Panel b**) shows the average eye movement traces for each participant in the target-face condition. The point marked ‘0’ on the x-axis denotes the onset of the critical faces presentation. Shaded regions around each line illustrate the standard error of the mean. In panel a), colored bars at the bottom highlight significant time intervals identified by the sample-by-sample linear mixed-effect regression analysis with FDR correction (*q* = .05, minimum cluster length = 200 ms). Specifically, the red bar indicates the interval where responses to the target face differed significantly from those to the averaged control faces. No significant intervals were observed for the familiar face condition.

As shown in Figure 3a, eye movements toward the target face were significantly larger than those toward control faces between 700–990 ms after stimulus onset (cluster length = 290 ms; median *β* = 35.5, peak *β* = 77.0, min FDR-*p* = 8.5 × 10^–23^). In contrast, no significant clusters were observed for familiar vs. control. These results indicate that participants reliably shifted gaze toward the target face, whereas gaze behavior for familiar faces did not differ from control faces, consistent with the task instructions.

Figure 3b shows the average eye movement distance for each participant in the target condition. Most participants exhibited a clear bump in gaze deviation beginning around 700 ms, peaking at approximately 990 ms, followed by a return toward baseline, with deviations generally within 100 px. In contrast, a subset of five participants displayed much larger deviations from the onset of the response, with distances exceeding 100 px and reaching up to nearly 900 px. These individuals appeared to adopt a different response strategy: rather than gradually following the target face with their gaze, they detected the target face and then made a direct saccade toward the edge of the screen in the target direction. Importantly, this strategy still ensured valid responses (defined as gaze deviations ≥ 50 px in the target direction from fixation) and was observed only in the target condition. It therefore does not affect the critical comparison between familiar and control faces.

**Individual level effect**. To detect familiar-control differences on eye movements at the individual level, we employed a leave-one-out analysis on two time windows identified in prior work (Ryan et al., 2007). That study found an initial eye movement towards familiar faces in the first second, followed by avoidance after one second when participants were shown multiple faces and instructed to avoid looking at the familiar ones. Based on this, we divided the trial into 0–1000 ms and 1000–2500 ms windows. To keep the predicted effect positive in both windows for ease of interpretation,, we subtracted control minus familiar in the first window (orienting phase) and familiar minus control in the second window (avoidance phase). This ensures that larger values consistently reflect stronger expected effects across both phases, avoiding the need for readers to reverse the sign when interpreting results.

For each individual, we identified two specific moments of the maximal eye movement difference in these respective directions based on the remaining 28 participants’ data. We then calculated the eye movement distance of the focal participant at these critical moments. One-tailed independent samples t-tests (*a* = .05) were then conducted to evaluate statistical significance of the differences observed in each time window for the focal participant.

**Figure 4:**
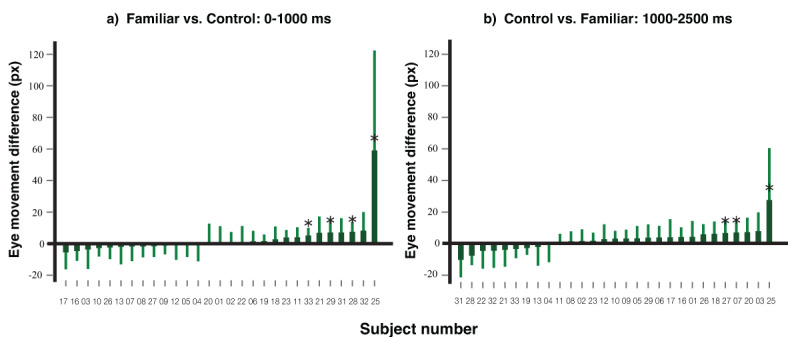
Individual Leave-one-out Analysis on Eye Movement Distance. *Note*. The eye movement differences of each participant towards the familiar face and average control faces in 0–1000 ms after the onset of the critical face are displayed in section **a)**, and the differences in eye movements towards the average control faces and familiar face in 1000–2500 ms are presented in section **b)**. Error bars represent the 95% confidence interval for each participant’s data. Participants whose differences reached statistical significance (*p* < .05) in the corresponding direction are denoted with an asterisk (*).

Figure 4 illustrates the directional differences for each participant within the first a) and second b) time windows. In both windows, the majority of participants displayed a trend consistent with the direction of the calculated differences, showing a more pronounced eye-position bias toward the familiar face when contrasted with the average control faces in the first second and away from the familiar faces after one second. This trend aligns with previous findings. Quantitatively, four participants showed a significant bias towards the familiar face within the first second, and three participants (including one who was also among the aforementioned four) showed a significant bias away from the familiar faces after one second.

#### Pupil size and rate of pupil size change

Based on pupil size data, we calculated the rate of change in pupil size by subtracting the previous sample from each subsequent sample on the pupil size trace. This change in pupil size was then smoothed with a 250-ms window while the original pupil size trace was not smoothed.

**Group level effect**. In line with the methodology applied to eye position, we implemented a sample-by-sample linear mixed-effects regression analysis to evaluate pupil size and pupil size change across the target face, the familiar face, and the averaged control faces (control 1 and control 2). The contrast between the familiar face and the averaged control faces was of primary interest, as it reflects the recognition-of-familiarity effect. For every 10 ms within the 2500 ms analysis window, condition was entered as a fixed effect, with the average of the two control conditions as the reference. To correct for multiple comparisons, *p*-values were adjusted using the Benjamini-Hochberg false discovery rate procedure (*q* = .05), and significant clusters were defined as ≥ 20 consecutive significant samples (≥ 200 ms).

**Figure 5:**
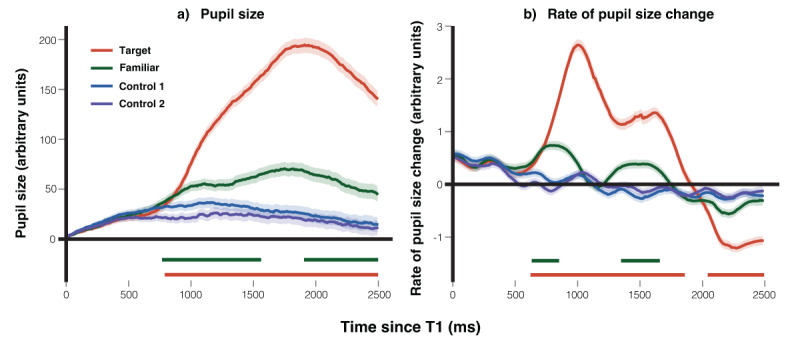
Average Pupil size and Rate of Pupil Size Change. *Note*. The graph displays average traces for a group of 29 participants over time. It features the target face (red line), the familiar face (green line), the control 1 face (blue line), and the control 2 face (purple line), showing the data for **a)** pupil size, and **b)** the rate of pupil size change. The colored bars represent significant time intervals where the data for the corresponding colored lines (conditions) differed significantly from the data for the averaged control conditions. The shaded regions around the lines illustrate the standard error of the mean.

As shown in Figure 5a, the pupil’s response to the target face was markedly larger than that to the control faces, spanning a sustained cluster from 820–2500 ms (median *β* = 137.3, peak *β* = 172.8, min FDR-*p* = 1.2 × 10^–20^). Notably, the pupil’s response to the familiar face also significantly exceeded that to the control faces, with two distinct clusters: 800–1570 ms (median *β* = 24.3, peak *β* = 39.4, min FDR-*p* = 4.8 × 10^–5^) and 1930–2500 ms (median *β* = 37.7, peak *β* = 45.1, min FDR-*p* = 2.3 × 10^–6^). The analysis results indicated no significant effects in the 1570–1930 ms window, despite clear differences visible in the figure. To investigate further, we conducted a z-value analysis, which revealed a sudden drop between 1570–1930 ms, indicating a potential convergence issue in the model. This issue likely reflects increased noise in the data during this time window. While simplifying the model by removing the random slope resolves the issue, we decided to retain the random slope, as this does not significantly affect the final conclusions.

Figure 5b illustrates the rate of pupil size change. Compared to control faces, the rate of dilation was significantly higher for the target face across a broad cluster from 650–1860 ms (median *β* = 1.43, peak *β* = 2.42, min FDR-*p* = 9.4 × 10^–36^), followed by a significant constriction cluster from 2060–2490 ms (median *β* = –0.88, peak *β* = –0.98, min FDR-*p* = 3.6 × 10^–15^). The familiar face also elicited significant increases in the rate of dilation relative to control faces, with clusters from 660–860 ms (median *β* = 0.72, peak *β* = 0.78, min FDR-*p* = 1.8 × 10^–6^) and 1370–1660 ms (median *β* = 0.51, peak *β* = 0.59, min FDR-*p* = 1.3 × 10^–6^).

**Supplementary Figure S1:**
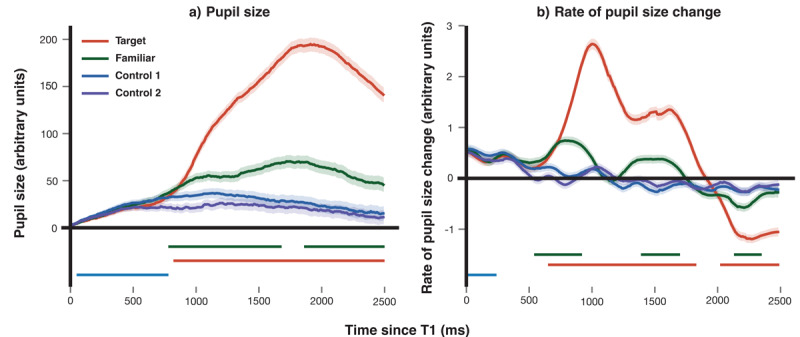
Average Pupil Size and Rate of Pupil Size Change in Experiment 1 (Models including Horizontal Gaze Position as a Covariate). *Note*. The figure displays group-average time courses (N = 29) for the target face (red line), familiar face (green line), control 1 (blue line), and control 2 (purple line), for (a) pupil size and (b) the rate of pupil size change. In this supplementary analysis, horizontal gaze displacement (x-position) was added as a covariate in the sample-by-sample linear mixed-effects models to control for potential effects of eye position on pupil measurements. Colored bars indicate significant intervals: red and green bars mark time windows in which the target or familiar face differed from the averaged control faces, and blue bars indicate intervals in which horizontal gaze displacement exerted a significant effect. Shaded regions reflect the standard error of the mean.

**Supplementary Figure S2:**
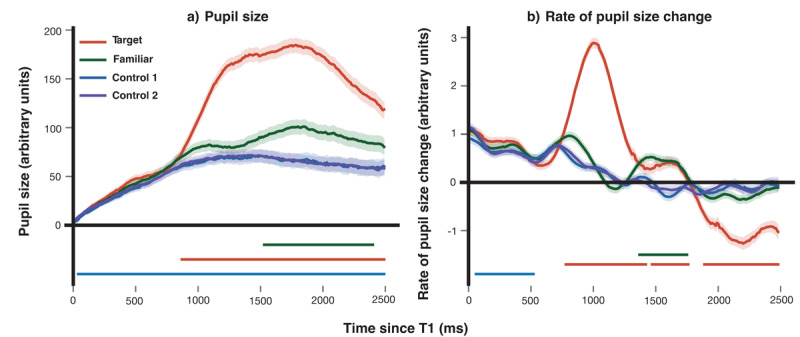
Average Pupil Size and Rate of Pupil Size Change in Experiment 2 (Models including Horizontal Gaze Position as a Covariate). *Note*. The figure shows group-averaged time courses (N = 27) for the target face (red), familiar face (green), control 1 (blue), and control 2 (purple), for (a) pupil size and (b) the rate of pupil size change. As in Experiment 1, horizontal gaze displacement (x-position) was included as a covariate in the sample-by-sample linear mixed-effects models to control for potential effects of eye position on pupil measurements. Colored bars indicate significant intervals: red and green bars mark time windows in which the target or familiar face differed from the averaged control faces, and blue bars indicate intervals in which horizontal gaze displacement exerted a significant effect. Shaded regions represent the standard error of the mean.

To rule out the possibility that pupil-size effects were influenced by horizontal gaze position, we re-ran all sample-by-sample linear mixed-effects regressions with horizontal eye position included as an additional covariate (see Appendix and Supplementary Figures S1–S2). These supplementary analyses showed that all key familiarity-related pupil effects remained intact, indicating that the main results cannot be attributed to differences in gaze position. Because including this covariate required trial-by-trial alignment of gaze and pupil samples, which led to the exclusion of additional trials with missing gaze data, the main analyses are reported using the full dataset without this covariate.

**Individual level effect**. We applied the leave-one-out approach to examine differences at the individual level for both pupil size and the rate of pupil size change. The procedural steps were identical to those used in the eye movement analysis in one direction, with the only difference being the substitution of eye-position difference between the familiar face and the control faces with differences pupil size and the rate of pupil size change. For the leave-one-out analysis, a one-tailed significance level of *p* < .05 was adopted as the threshold for determining statistical significance.

**Figure 6:**
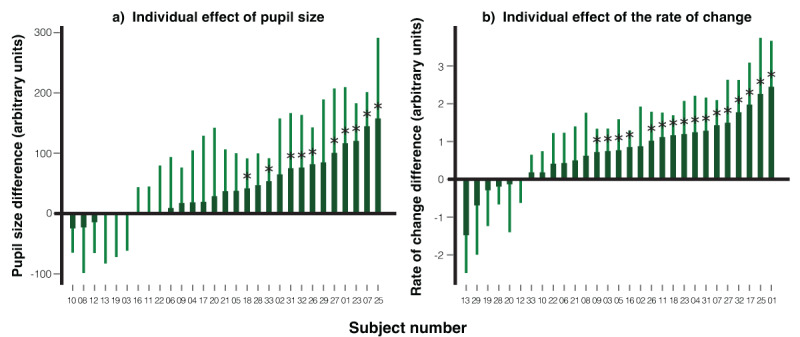
Individual Leave-one-out Analysis on Pupil Size and Rate of Pupil Size Change. *Note*. The chart illustrates the difference between responses to the familiar face and the average of the control faces for each participant in terms of **a)** mean pupil size, and **b)** mean rate of pupil size change. For each participant, the comparison point was determined by the moment where the response to the familiar face was the largest compared to the averaged control faces, based on data from the other 28 participants. Participants with significant differences in their responses are highlighted with an asterisk (*). Error bars represent the 95% confidence intervals for each individual’s measurements.

As shown in Figures 6, representing pupil size (a) and the rate of pupil size change (b) respectively, most participants exhibited a larger response to the familiar face compared to the averaged control faces (control 1 and control 2). Statistical analysis revealed that 10 participants showed a significant difference in pupil size, while 16 participants demonstrated a significant difference in the rate of pupil size change.

### Discussion

When participants were instructed to use eye movements to respond by following the target face, they did so reliably. Importantly, contrary to our expectations, when a familiar face was presented, participants were able to maintain gaze control, and no significant group-level eye movement differences between familiar and control faces were observed. At the individual level, only a few participants showed notable deviations in gaze behavior. By contrast, the pupil measures revealed robust effects of familiar faces. Compared to control faces, familiar faces elicited significantly greater pupil dilation from 800–1570 ms and 1930–2500 ms, and a faster rate of pupil size change from 660–860 ms and 1370–1660 ms. At the individual level, significant differences were found in 10 out of 29 participants (34.5%) for pupil size, and in 16 participants (55.2%) for the rate of pupil dilation. These results suggest that pupil dynamics provide a more sensitive index of familiar-face recognition than eye movement measures in this task.

## Experiment 2

To build upon the findings from Experiment 1, we designed Experiment 2 to examine whether removing the oculomotor constraints would change the balance between orienting and suppression-related responses to familiar faces. By allowing participants to move their eyes freely, we aimed to test whether gaze measures, particularly differences between familiar and control faces, would emerge more strongly in the absence of enforced fixation, while still assessing pupil-based indicators of familiarity recognition.

### Method

#### Participants

Initially, 31 participants were recruited from the first-year participant pool or the paid-participant pool of the Psychology Department of the University of Groningen. They participated in the experiment in exchange for either course credits or monetary compensation. A response was counted as correct if participants pressed the ‘yes’ key in target trials or ‘no’ key in non-target trials. We used ~70% accuracy as a pragmatic reference point to ensure that participants were able to perform the task at a basic level. Two participants whose overall accuracy fell well below this level were excluded. In addition, two other participants were excluded because of technical problems that resulted in fewer than 100 usable trials out of 192. The data of the remaining 27 participants (mean age: 21.1 years, range: 17–32 years, 21 females) were analyzed. All participants signed up voluntarily and written informed consent was obtained prior to participation from both the participants and their parents, who provided us with an image of themselves to be used as the familiar faces. All participants reported normal or corrected-to-normal visual acuity and none of them reported color-blindness. They were required to sleep well the night before and not wear any eye makeup. The study was conducted in accordance with the World Medical Association Declaration of Helsinki (2013) and approved by the ethics committee of the Psychology Department of the University of Groningen (approval number: PSY-2023-S-0283).

#### Differences from Experiment 1

This experiment followed the same materials, equipment, design, and procedure as the previous one, only with two key differences: 1). Participants were no longer required to fixate continuously on the fixation dot at the center of the screen until the target face was detected; instead, they were free to move their eyes. 2). Participants were not required to respond using eye movements immediately after they detected a target face. Instead, participants responded using a keyboard after the entire sequence of faces and an additional 2000 ms of fixation dot presentation. They answered whether there was a male face when the familiar face was the mother’s, or whether there was a female face when the familiar face was the father’s. The corresponding answers of the two keys, ‘c’ and ‘m’, were assigned for ‘yes’ or ‘no’ based on the participant number’s parities and were counterbalanced.

#### Preprocessing of eye-tracking data

The preprocessing of eye-tracking data in this experiment was identical to that of the previous experiment. There were a total of 5184 trials. Out of these, 215 trials were excluded because the baseline pupil size was undefined or the baseline pupil size exceeded 2 standard deviations from the mean baseline pupil size.

#### Transparency and Openness

The stimuli (images of familiar faces excluded) and data for the experiment are available on the OSF: https://osf.io/sk5zq/.

### Results

#### Behavioral data

Participants indicated whether a target face was present by pressing a button after the trial. Accuracy rates were high across all conditions. For target trials, mean accuracy was 91.6% (range: 77.1–100.0%). For familiar-face trials, mean accuracy was 88.6% (range: 56.2–100.0%), while Control 1 and Control 2 yielded average accuracies of 90.6% (range: 64.6–100.0%) and 88.8% (range: 56.2–100.0%), respectively. Again paired t-tests showed that there was no significant difference between accuracy in the familiar condition and the average of two control conditions (*t*(26) = -1.804, *p* = 0.0813).

Compared to Experiment 1, where the target condition yielded a mean accuracy of just 39.1%, the marked improvement in Experiment 2 suggests that participants were able to reliably detect the target face when not constrained to respond via eye movements. In Experiment 1, the requirement to maintain central fixation except when detecting the target may have prevented participants from confirming each face individually, thereby limiting detection opportunities. Thus, the lower accuracy in Experiment 1 may reflect both the motor demands of executing gaze shifts and the cognitive challenge of recognizing and responding to the target under restricted viewing conditions.

**Figure 7:**
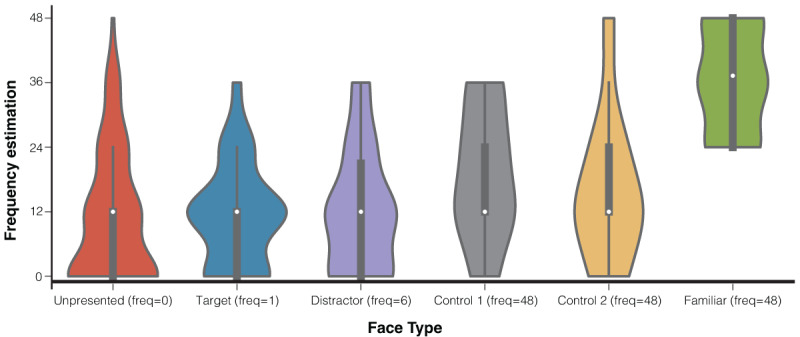
Frequency Estimation in the Memory Task. *Note*. Each color corresponds to a type of face, with the corresponding types and actual frequencies marked on the X-axis. The vertical black lines in each violin represent the specific estimates given by the participants for the frequency of occurrence of this type of face, while the white dots show the average value.

After the experiment concluded, we also arranged a memory test similar to the one in Experiment 1, where participants were asked to estimate the frequency of occurrence for 11 faces (details can be found in the procedure section of Experiment 1). Consistent with the results of Experiment 1, all participants reported having seen the familiar face, and they estimated a higher frequency of occurrence for the familiar face compared to their estimates for other types of faces, as displayed in Figure 7.

#### Eye movement

The analysis of eye movement data for this experiment was conducted using the same methods as those applied in Experiment 1.

**Group level effect**. A sample-by-sample linear mixed effects regression analysis was conducted on eye movement distances in response to the target face, the familiar face, and the average of the two control faces, using the same procedures as in Experiment 1. To correct for multiple comparisons, *p*-values were adjusted using the Benjamini-Hochberg false discovery rate procedure (*q* = .05), and significant clusters were defined as ≥ 20 consecutive significant samples (≥ 200 ms).

**Figure 8:**
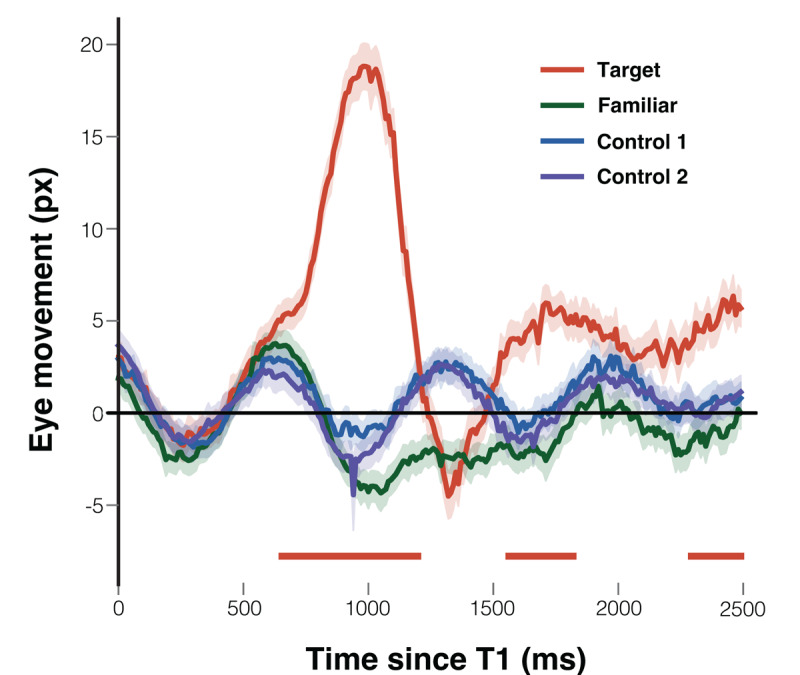
Average Eye Movement Distances. *Note*. The graph illustrates the average eye movement distances across time for various face types: the target face (red line), familiar face (green line), control 1 face (blue line), and control 2 face (purple line). The ‘0’ point on the x-axis denotes the onset of the critical face presentations. The shaded areas surrounding each line indicate the standard error of the mean. Colored bars at the bottom of the graph indicate significant time intervals identified by the sample-by-sample linear mixed-effect regression analysis with FDR correction (*q* = .05, minimum cluster length = 200 ms). Specifically, the red bars denote intervals where eye movements in response to target faces differed significantly from those to the averaged control faces.

As depicted in Figure 8, eye movements in response to the target in Experiment 2 were less pronounced than those in Experiment 1, with the peak eye movement in Experiment 2 reaching ~20 px, compared to ~100 px in Experiment 1. Eye movements toward both control and familiar faces followed the rhythmic motion of the images, alternating direction approximately every 300 ms.

The regression analysis revealed significant tracking of the target face relative to the control faces across three distinct time windows: 630–1190 ms (median *β* = 12.8, peak *β* = 20.8, min FDR-*p* = 4.0 × 10^–11^), 1550–1820 ms (median *β* = 5.2, peak *β* = 6.2, min FDR-*p* = 1.1 × 10^–4^), and 2290–2500 ms (median *β* = 4.6, peak *β* = 5.7, min FDR-*p* = 7.2 × 10^–5^). In contrast, no significant clusters were found for familiar vs. control, indicating that participants’ gaze behavior toward familiar faces did not differ systematically from that toward control faces.

**Figure 9:**
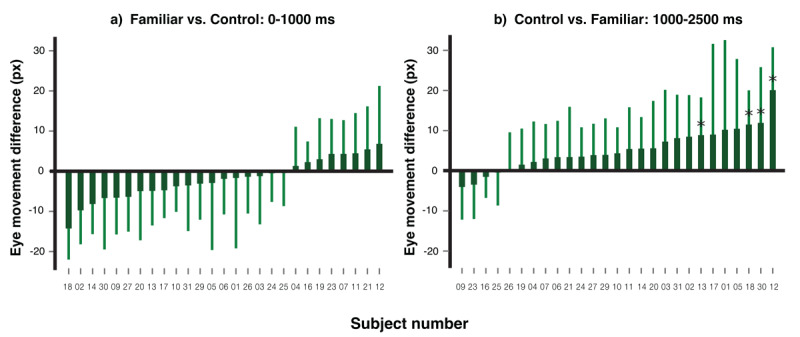
Individual Leave-one-out Analysis on Eye Movement Distance. *Note*. The eye movement differences for each participant, comparing the familiar face to the average control faces within the first 0–1000 ms after the critical face’s onset, are shown in **a)**. The eye movements differences directed toward the average control faces compared to the familiar face in the subsequent time window of 1000–2500 ms are shown in **b)**. Error bars have been included to depict the 95% confidence intervals. Those participants whose eye movement differences were significant in the designated direction are marked with an asterisk (*).

**Individual level effect**. Figure 9 shows, for each participant, the differential eye movements between the familiar and control faces within the initial 0–1000 ms time window in a), and the contrast in eye movements between the control and familiar faces in the subsequent 1000–2500 ms time window in b). The data for each individual has been marked to reflect the outcomes of the leave-one-out analysis, which considered the corresponding directions of eye movement. The majority of participants had reduced eye movement towards the familiar face relative to the averaged control faces in both time windows. From a statistical perspective, no one showed a bias in the 0–1000 ms time window and four participants showed a statistically significant bias in the direction opposite to the familiar face in comparison to the averaged control faces in the 1000–2500 ms time window.

#### Pupil size and rate of pupil size change

The analysis of pupil data for this experiment was conducted using the same methods as those applied in Experiment 1. To correct for multiple comparisons, *p*-values were adjusted using the Benjamini-Hochberg false discovery rate procedure (*q* = .05), and significant clusters were defined as ≥ 20 consecutive significant samples (≥ 200 ms).

**Figure 10:**
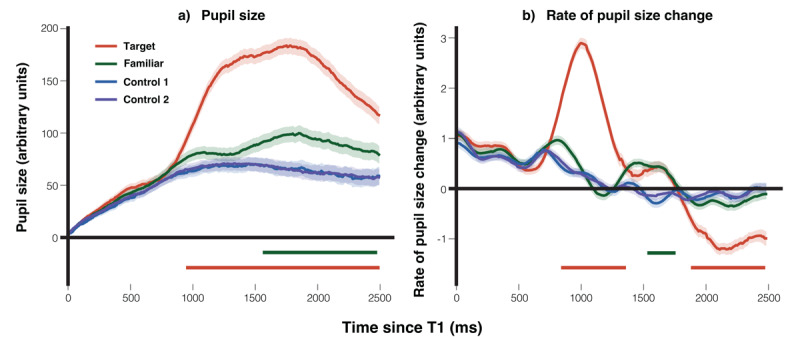
Average Pupil size and Rate of Pupil Size Change. *Note*. The chart presents the average pupil traces of over time for 27 participants. It shows the responses to the target face (indicated by a red line), familiar face (green line), control 1 face (blue line), and control 2 face (purple line) with regards to **a)** pupil size, and **b)** the rate of pupil size change. The shaded areas surrounding each line represent the standard error of the mean. Colored bars highlight the significant time periods where the responses to the corresponding conditions significantly diverged from those to the averaged control conditions.

**Group level effect**. As demonstrated in Figure 10a, the pupil’s response to the target face was significantly larger than that to the control faces across a broad and sustained cluster from 880–2500 ms (median *β* = 97.5, peak *β* = 118.5, min FDR-*p* = 2.1 × 10^–16^). The familiar face also elicited significantly larger pupil responses than the control faces, with a cluster from 1530–2480 ms (median *β* = 29.2, peak *β* = 34.9, min FDR-*p* = 9.9 × 10^–5^). Figure 10b illustrates the rate of pupil size change. Compared to control faces, participants exhibited a significant increase in the rate of dilation for the target face from 780–1310 ms (median *β* = 1.84, peak *β* = 2.58, min FDR-*p* = 3.5 × 10^–50^), followed by a significant constriction cluster from 1880–2490 ms (median *β* = -0.92, peak *β* = –1.15, min FDR-*p* = 1.5 × 10^–13^). For the familiar face, the rate of dilation was significantly greater than for the control faces during 1510–1730 ms (median *β* = 0.63, peak *β* = 0.67, min FDR-*p* = 5.6 × 10^–7^).

**Figure 11:**
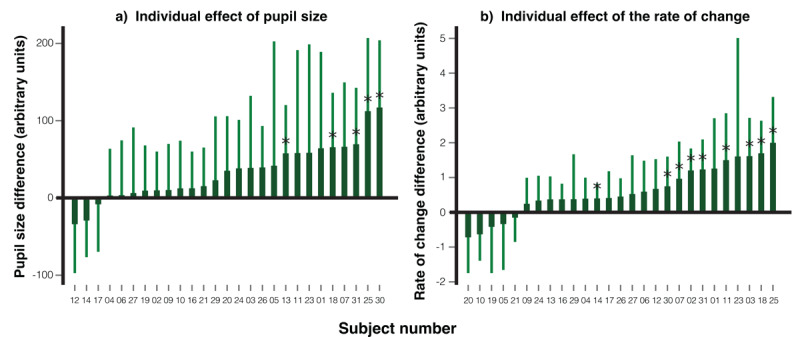
Individual Leave-one-out Analysis on Pupil Size and Rate of Pupil Size Change. *Note*. The differences in responses for each participant (*n* = 27) when comparing the familiar face with the average of the control faces are presented in terms of **a)** mean pupil size, and **b)** mean rate of pupil size change. The specific moment chosen for comparison for each participant was the one where the difference in response to the familiar face was at its peak compared to the averaged control faces, as determined by the data from the other 26 participants. Those participants whose response differences reached statistical significance are marked with an asterisk (*). Error bars are included to denote the 95% confidence intervals.

**Individual level effect**. The analysis of both pupil size and the rate of pupil size change at the individual level in this experiment mirrored that of Experiment 1. Figure 11a illustrates each participant’s pupil size difference, while Figure 11b illustrates the rate of pupil dilation change, when viewing the familiar face versus the average control faces. Qualitatively, a majority of the participants showed increased pupil dilation and a quicker dilation rate in response to the familiar face compared to the averaged control faces. The leave-one-out analysis indicated that, of the 27 participants, 5 exhibited statistically significant differences in pupil size and 9 in the rate of pupil dilation.

### Discussion

Contrary to the first experiment, in which participants were instructed to keep fixating a central dot except when the target face appeared, in the second experiment they actively followed the target face. The group-level eye movement analysis revealed robust tracking of the target face, with no significant differences between familiar and control faces. By contrast, the pupil results consistently showed enhanced responses to familiar faces, in line with Experiment 1. Specifically, familiar faces elicited larger pupil size (1530–2480 ms) and a transient increase in the rate of dilation (1510–1730 ms) relative to control faces, both of which are consistent with the recognition-of-familiarity effect central to this study. At the individual level, significant differences were observed in 5 out of 27 participants (18.5%) based on pupil dilation, and in 9 participants (33.3%) for the rate of dilation.

## Interactions Between Experiment Designs and Conditions

To examine whether the two experimental designs (constrained-gaze with eye-movement responses vs. free-gaze with keyboard responses) differentially modulated the responses to target and familiar faces relative to control faces, we conducted sample-by-sample linear mixed-effects model analyses with eye movement, pupil size, and rate of pupil size change as dependent variables. Experimental conditions (average control as reference, target, familiar), experiment design (constrained-gaze vs. free-gaze), and their interaction were included as fixed effects, with random slopes for experimental conditions within subjects. Statistical significance was determined by Benjamini-Hochberg FDR correction (*q* = .05) and a minimum cluster length of 200 ms.

### Eye movement

For gaze position, no significant main effect of experimental design was observed. Moreover, there were no significant interactions with either familiar or target faces. This indicates that the two experimental designs did not differentially modulate eye movement responses to faces.

### Pupil size

The two different experimental designs demonstrated a main effect on pupil dilation for average control faces across an extended interval 730–1750 ms (len = 1020 ms; median *β* = 19.79, peak *β* = 22.10; min FDR-*p* = .0047), indicating overall larger pupil responses in the free-gaze task than in the constrained-gaze task. In addition, a significant interaction between experiment type and target faces was observed from 2030–2500 ms (len = 470 ms; median *β* = –34.68, peak *β* = –35.94; min FDR-*p* = .00074), indicating that the target-control difference was amplified in the constrained-gaze task compared to the free-gaze task. There was no interaction effect between familiar faces and experiment design.

A possible explanation of this main effect of type and the interaction pattern is that in Experiment 2, participants were free to move their eyes, which likely increased the viewing time for each stimulus face, resulting in greater overall pupil dilation. In principle, this should also have led to larger pupil responses to both target and familiar faces in Experiment 2. However, the higher task demands and oculomotor constraints in Experiment 1 appear to have amplified pupil responses to targets, reversing this pattern for the target-control comparison, while the familiar-control difference was effectively offset, leading to no observable interaction for familiar faces.

### Rate of pupil size change

The two different experimental designs showed a main effect on the rate of pupil size change for average control faces from 540–840 ms (len = 300 ms; median *β* = 0.283, peak *β* = 0.353; min FDR-*p* = 1.8 × 10^–5^), with faster dilation in the free-gaze task than in the constrained-gaze task. Moreover, a significant interaction between experiment type and target faces was observed from 1360–1830 ms (len = 470 ms; median *β* = –0.440, peak *β* = –0.569; min FDR-*p* = .00035), with greater target-control differences in the constrained-gaze task than in the free-gaze task. No significant interaction with familiar faces was found.

To verify whether the null interaction for familiar faces could be explained by changes in the familiar condition itself, we conducted an additional reverse-coding analysis with familiar faces as the reference category. This analysis revealed no significant clusters for either pupil size or pupil size change, indicating that pupil responses to familiar faces did not differ systematically between the two experimental designs.

## General Discussion

Across two experiments, we tested whether task-irrelevant familiar information can be detected in the moving serial visual presentation (MSVP) paradigm using eye movement and pupil-based measures. The key finding is that requiring participants to maintain central fixation and respond to the targets with eye movements enhanced the sensitivity of pupil-based measures for detecting task-irrelevant familiarity, whereas direct eye-movement responses contributed little to the detection of familiar faces.

In both task designs, in which participants were either required to keep their gaze fixed at the center of the screen, using only eye movements to follow the target face for their response (a constrained-gaze design with eye-movement responses, Experiment 1), or were allowed to freely observe each facial stimulus and then respond using a keyboard (a free-gaze design with keyboard responses, Experiment 2), showed that familiar faces elicited larger pupil dilation and a faster rate of dilation than control faces. At the individual level, detection rates based on the rate of pupil size change (55.2% in Experiment 1, 33.3% in Experiment 2) exceeded those based on pupil size (34.5% in Experiment 1, 18.5% in Experiment 2). This pattern is consistent with the finding of our previous RSVP-CIT study (Chen, Büchel, et al., 2023), in which the rate of pupil size change proved more sensitive than pupil size itself.

Furthermore, although interaction analyses indicated that the differential pupil responses to familiar versus control faces were comparable across the two experimental designs, the individual-level results revealed higher detection rate in the constrained-gaze design (55.2%) than in the free-gaze design (33.3%). This suggests that constraining eye movements not only increased task difficulty (a point we return to later) but also amplified the diagnosticity of pupil-based measures of familiarity. Importantly, the 55.2% individual detection rate is markedly higher than the 22.6% detection rate for familiar faces previously observed in RSVP-CIT using the same measure (Chen, Büchel, et al., 2023). Thus, it suggests that this new paradigm with the eye-movement suppression requirement appears to enhance sensitivity to task-irrelevant familiarity signals at the individual level than the ‘classic’ RSVP-based CIT.

However, it should be noted that RSVP-based CIT paradigms with EEG have reported substantially higher individual detection rates, suggesting that while our MSVP approach shows promise, further refinement is needed before it can match the sensitivity levels achieved with EEG. A crucial next step for assessing the applied utility of this paradigm and enabling direct comparison with previous studies will be to include an innocent group and implement ROC/AUC analyses.

Our interaction analyses shed further light on how task design influenced pupil responses. In the control condition, pupil responses were consistently larger in the free-gaze design than in the constrained-gaze design, suggesting that the longer effective viewing time afforded by free eye movements may enhance overall pupil dilation. By contrast, target-control differences were significantly stronger in the constrained-gaze design, likely reflecting the heightened oculomotor control demands when participants had to suppress eye movements and then rapidly saccade to the target once detected. For familiar faces, no systematic differences emerged between the two designs. Reverse-coding analyses confirmed that familiar responses themselves did not differ significantly across tasks. One possible interpretation is that both designs may have led to comparable increases in pupil responses, driven by longer viewing time in the free-gaze task and by greater cognitive control in the constrained-gaze task. However, this account remains tentative and requires further empirical validation.

We found that eye movements played a limited role in detecting processing of familiar faces within this paradigm. Contrary to our expectations, when participants were instructed to fix their gaze at the center of the screen, the moving familiar faces did not reliably induce uncontrollable eye movement following these stimuli. Participants were able to effectively control their eye movements, with no significant differences observed at the group level between familiar and control faces. This finding partially aligns with one of the three experiments from Lancry-Dayan et al.’s study (2018). In that experiment, participants were instructed to use countermeasures, specifically to tightly control their eye movements to avoid detection of concealed information. As a result, initial gaze towards familiar faces was not observed in 0–2500 ms. However, unlike that experiment, where gaze avoidance for familiar faces eventually emerged after 2500 ms, our results did not show any gaze avoidance for familiar faces throughout. This could be related to our very short SOA (300 ms) and the trial duration of only 2500 ms.

We also found that the accuracy for detecting target faces was markedly lower in the constrained-gaze design (39.1%) than in the free-gaze design (91.6%), indicating that the former imposed greater task difficulty. Given the absence of significant eye-movement differences between familiar and control faces in both experiments, this increased difficulty likely arose from factors such as the inability to improve stimulus inspection through eye movements, reduced viewing time for each stimulus, and the need to quickly saccade to and then track the target face once detected. Such heightened cognitive control, similar to that induced by countermeasures or concealment instructions, may occupy attentional resources and produce stronger pupil responses to familiar faces. Further research is needed to directly test these potential commonalities. The present combined approach of using both pupil size and eye movement measures to index cognitive effort may also be applicable to other cognitive paradigms, where it could prove even more effective and offer novel insights.

It is crucial that when applying this paradigm in practical settings, its ability to withstand countermeasures also needs to be tested. Unlike the RSVP-CIT, which presents each stimulus for about 100 ms, this paradigm requires a longer presentation time to allow for stimulus movement and for participants to follow it with eye movement, like the 300-ms SOA used in our study. A longer SOA might allow participants to recognize control faces and intentionally increase their eye movement following these faces. As a result, their cognitive control might be enhanced, reducing the difference of pupil size change between control and familiar faces. Moreover, a type of deliberate strategy, treating a control face as if it were familiar, represents a plausible confound in applied use and should be explicitly examined. Specific testing of countermeasures resistance will need to be conducted in subsequent research.

To conclude, the current novel paradigm for detecting task-irrelevant familiar information, combining moving serial visual presentation (MSVP), shows higher sensitivity compared to the previous RSVP-CIT study, detecting more familiar faces at an individual level. Importantly the constrained-gaze version of MSVP, in which participants were required to suppress eye movements except toward targets, proved more sensitive than the free-gaze version. Even though direct eye movements are not an effective measure in this paradigm, pupil size, especially the rate of change in pupil size, proved to be a valid index of familiarity. However, current detection rates are still far from the near-perfect levels required for applied use. Further work should investigate the internal cognitive mechanisms underlying pupil size change, test the paradigm’s resistance to countermeasures, and incorporate innocent participants to allow ROC/AUC-based evaluation.

## Data Accessibility Statement

All materials, anonymised data, and analysis scripts relevant to the reported findings are available via the Open Science Framework (OSF). Experiment 1 materials and scripts can be accessed at https://osf.io/ske56/, and Experiment 2 materials and data at https://osf.io/sk5zq/. Familiar face images are excluded from public sharing to ensure participant and parental confidentiality.

## Appendix

### Control analysis: Pupil models including horizontal gaze position

To rule out the possibility that pupil-size effects were confounded by differences in horizontal gaze position, we re-ran the sample-by-sample linear mixed-effects regression analyses for both pupil size and the rate of pupil-size change in both experiments, this time including horizontal gaze displacement (x coordinate) as an additional covariate. In these models, condition (target, familiar, averaged control) was entered as a fixed effect, with horizontal gaze displacement added as a second fixed predictor; random slopes for condition were specified within participants.

The results for Experiment 1 and 2 are shown in Supplementary Figures S1 and S2, respectively. Across both experiments, adding horizontal gaze displacement did not meaningfully alter the effects of familiarity. In Experiment 1, the familiar-control differences in both pupil size and the rate of pupil change remained present in virtually the same time windows as in the analyses without gaze included, demonstrating that the familiarity effects are not driven by differences in eye position. Horizontal gaze displacement itself showed significant effects only in an early portion of the time series for pupil size change, and a broader but functionally unrelated interval for pupil size, neither of which overlapped with the intervals most diagnostic of familiarity. In Experiment 2, horizontal gaze displacement for pupil size exhibited wider clusters of significance, which is consistent with participants being free to move their eyes, yet even in this case, the familiarity effects reflect genuine pupillary responses rather than artifacts of horizontal eye movements.
